# Internet Use and Effects on Mental Well-being During the Lockdown Phase of the COVID-19 Pandemic in Younger Versus Older Adults: Observational Cross-Sectional Study

**DOI:** 10.2196/46824

**Published:** 2024-02-06

**Authors:** Chou Chuen Yu, Nien Xiang Tou, James Alvin Low

**Affiliations:** 1 Geriatric Education and Research Institute Singapore Singapore; 2 Khoo Teck Puat Hospital Singapore Singapore

**Keywords:** COVID-19, digital divide, well-being, older adults, information and communication technology, internet of things, online, mental health, lockdown, depression, stress, anxiety, digital technology, pandemic

## Abstract

**Background:**

Majority of individuals, including both younger and older adults, had to adapt to digital means to cope with lockdown measures and pandemic-induced lifestyle changes during the COVID-19 pandemic. While internet accessibility was beneficial during the pandemic, existing literature suggests that excessive use could lead to the rise of problematic internet use in adolescents and younger adults. However, the effects on older adults remain unclear.

**Objective:**

This study aimed to examine differences in internet use during the lockdown phase of the COVID-19 pandemic and explore how age differences in mental health could be explained by time spent on the internet.

**Methods:**

A door-to-door survey of a nationally representative sample of 602 adults in Singapore was carried out using computer-assisted personal interviewing during the early phase of the COVID-19 pandemic (October to November 2020). Participants were categorized into younger (21-59 years old) and older (60 years or above) age groups. We assessed self-reported measures of depression, anxiety, and stress; psychosocial adaptability; ability to perform essential activities; social support; health status; digital media use patterns, and time spent on the internet. Procedures complied with existing safe distancing measures.

**Results:**

Older adults reported being less able to use digital platforms to meet needs and acquire information updates compared with younger adults during the lockdown period of the pandemic. Older adults spent significantly less time on the internet for both work and personal uses per day (mean 146.00 min, SD 9.18 min) compared with younger adults (mean 433.27 min, SD 14.32 min). Significant age differences in depression, anxiety, and stress were found, with younger adults showing poorer mental health. Mediation analysis showed that age differences in depression, anxiety, and stress were partially explained by time spent on the internet. These variables together explained 43%, 40%, and 40% of the variances in depression, anxiety, and stress scores, respectively.

**Conclusions:**

The findings showed that younger adults spent significantly more time on the internet compared with older adults during the lockdown phase of the pandemic. They were also ahead in their ability to use digital resources to meet needs and engage socially compared with older adults. Despite this, the mental health of younger adults was poor, and this was partially accounted for by the amount of time spent on the internet. Since past research suggests that excessive time spent on the internet could lead to disordered use, the benefits brought by digital technologies could have been attenuated during the lockdown phase of the pandemic. Considering this potential negative effect, it is imperative to educate both young and old adults in the appropriate use of information and communication technology.

## Introduction

COVID-19 has brought about significant changes to the lives of people, with negative impacts on well-being. During the initial stages of the COVID-19 pandemic, “spatial distancing” was a promoted practice that entailed keeping a safe distance between individuals and reducing the number of close interactions individuals have with one another [1]. In a bid to protect lives from this pandemic, many governments around the world subsequently proceeded to initiate lockdowns that mandated the restriction of people’s movements and confined citizens to their homes, limiting, if not halting, unnecessary interactions [2]. Even after lockdowns, various forms of regulations and recommendations on spatial distancing were maintained. As a consequence of such measures, stress and anxiety greatly affected individuals, families, and the society as a whole [3-5]. Even in late 2022, lockdown measures had not been fully discarded and continued to be practiced in some countries [6]. A common trend across the world was that time spent isolated at home increased significantly for most individuals with a resultant loss of daily routines [2]. Even when mandatory movement restrictions were lifted, different waves of new COVID-19 infections meant that people often found themselves being confined to their homes owing to stay-at-home orders or self-sequestration [7]. With most physical activities being curtailed, running activities online became the new normal.

The use of information and communication technology (ICT) was critical during the COVID-19 crisis. ICT not only allowed for the dissemination of timely COVID-19–related information to the public to act upon, but also made it possible to work and study remotely. Arguably, the psychological impact of isolation was mitigated by ICT as friends and families were kept connected despite the physical restrictions imposed by lockdowns [8]. ICT provided access to various forms of entertainment and even materials guiding physical exercises [9]. Access to entertainment through ICT is important as it can help to alleviate the stress of daily living [8,10]. Although a growing number of older adults have been adopting ICT [11,12], the digital exclusion of older adults, otherwise known as the “grey digital divide,” has been an ongoing global issue during the pandemic [13,14]. Older adults face problems in a variety of basic tasks (eg, booking of tickets, claiming benefits online, and gaining access to health care services through appointments) and face exclusion because they cannot connect with their peers through online platforms owing to limited digital skills [14]. Because of the effects of restrictions in social gatherings and spatial distancing during the pandemic, this grey digital divide puts older adults at a disadvantage and may lead to feelings of social isolation and possibly exacerbate health disparity among older adults [15].

While the benefits of ICT during the pandemic seem apparent, especially if used for the purpose of communication, information, and task performance [7,16], excessive use of ICT can be problematic. For instance, the use of ICT without moderation for online gambling, viewing pornography, playing video games, viewing social media, and shopping may lead to higher risks of disordered use [2,17,18]. Research has shown that the disordered use of the internet, also known as problematic internet use, can cause emotional distress and significantly affect different domains in one’s life, including personal, family, and social relations. It can also lead to adverse effects on work or education and other areas of functioning [2,18]. In the current COVID-19 pandemic, an increase in problematic internet use has been reported and excessive internet use has been suggested as a means to cope with the enforced sedentary norms [19,20] in part due to lockdown measures and possibly pandemic-induced life changes [21,22]. The negative consequences of excessive internet use on mental health have been reported in studies during the current pandemic [23,24]. Research conducted around the same time as the inception of this study has shown that there was an increase in problematic internet use during the COVID-19 pandemic [25-32]. However, other than the studies conducted in Taiwan [28] and Japan [29], most of these studies adopted a convenience sampling approach in data collection, and the inferences made from these studies could be limited owing to the sampling bias and poor generalizability [33] associated with this sampling approach. Moreover, the populations of interest in most of these studies were younger adults and adolescents. A recent review on problematic internet use during the COVID-19 pandemic affirmed our aforementioned observation [34]. The authors of the review called for future studies comparing age influences. Only the study carried out in Japan by Oka et al [29] examined age differences. The study found that internet gaming–related problems in younger adults (<30 years) increased during the pandemic, and the numbers were much higher than those for more mature adults. Properly sampled studies comparing the effects of internet use on well-being between younger and older adults were largely missing during the lockdown period of the pandemic.

In view of the identified gaps, this study aimed to examine (1) differences in internet use between younger and older adults during the lockdown phase of the COVID-19 pandemic, and (2) the influence of internet use on the relationship between age and mental health. Given what we know about the internet use of younger adults during the pandemic, we hypothesized that younger adults would have greater use of the internet as compared with older adults, and such a difference in digital use would affect the relationship between age and mental health. This study was conducted in Singapore, an island country and city-state in maritime Southeast Asia. According to official statistics [35], internet use in Singapore has increased from 58% in 2019 to 81% in 2021 among residents aged 60 years or older. The findings from this study may contribute to our understanding of the role of internet use for mental health issues among older adults in countries with increasing internet adoption rates.

## Methods

### Study Design, Setting, and Participants

This study employed an observational cross-sectional study design. Residents aged 21 years or above were recruited using stratified random sampling with stratification based on housing type, geographical region, gender, and age group. A door-to-door survey was conducted by experienced interviewers between October 17, 2020, and November 27, 2020, not long after partial lockdown restrictions were gradually lifted [20]. The in-person surveying approach ensured inclusion of study participants who might not have access to the internet to mitigate selection bias. The questionnaire administered by the interviewers was worded in the English language, which is the main language in Singapore. To be included in the study, participants were required to have resided in Singapore during the lockdown phase of the pandemic (known locally as the circuit breaker (CB) period between April 7, 2020, and June 1, 2020), in which spatial distancing measures were imposed (known locally as safe distancing). They were also required to be able to speak English and be aged 21 years or older. Interviewers were trained by a geriatrician to exclude older adults who exhibited possible signs of cognitive impairment (eg, drowsiness, agitation, and incongruent language) during the process of taking informed consent. Additionally, among those aged above 70 years, the Abbreviated Mental Test (AMT) [36] was used to screen for possible signs of dementia and other cognitive impairments, and participants were excluded if they failed 1 of the 3 items in the AMT. Safe distancing rules were adhered to during the data collection period.

### Ethical Considerations

Ethics approval was obtained from the National Healthcare Group Domain Specific Review Board (2020/00973), and written informed consent was obtained from all participants. Participants were reimbursed with grocery vouchers worth SGD 10 (USD 7.50) for participation. All data collected were deidentified prior to analysis.

### Survey Measures

#### Digital Platform Use

Digital platform use was a single item measure. Participants responded to the question “What did you use digital media for during the CB [circuit breaker] period?” Participants selected from a list of 7 common uses: (1) food delivery, (2) online banking, (3) grocery shopping, (4) online shopping (excluding groceries), (5) online entertainment, (6) social media, and (7) online telecommunication. Participants could also specify, in free-text format, other uses beyond the 7 listed if required. The option “did not use” was available if participants did not make use of any of the platforms.

#### Perform Essential Activities

The ability to perform essential activities was measured using a 6-item measure. The individual items have been reported in the Results section given the focus on the activities related to internet use (eg, “I was able to use online services to settle what I needed to do [eg, online banking and filling application forms],” “I was able to use telecommunication platforms for work or education purposes,” and “I was able to use online platforms to obtain my supplies [eg, groceries and buy take outs] whenever there was a need to”). Participants responded on a 5-point Likert scale ranging from 1 (strongly disagree) to 5 (strongly agree). The scale has good internal consistency (α=.81).

#### Use of the Internet Before and After the Pandemic

Use of the internet before and after the pandemic was determined by whether participants reported the use of any of the 9 online services commonly used in the local setting (eg, Redmart, Shopee, Taobao, FairPrice Online, Foodpanda, Grabfood, Cold Storage online, Deliveroo, and Lazada) before as well as after or during the pandemic. Participants could also report any other platforms that they used, which were not listed as options in the survey, in free-text format.

#### Time Spent on the Internet

Time spent on the internet was measured using the following question that was asked in the context of the pandemic: “On average, how much time do you spend on the internet per day (for both work and personal uses)?” Participants reported the number of hours and minutes.

#### Mental Health

Mental health status was measured using the shortened version of the Depression, Anxiety, and Stress Scale (DASS-21) [37]. The DASS-21 consists of three 7-item subscales designed to measure levels of depression (α=.87), anxiety (α=.76), and stress (α=.87). A sample item for depression is as follows: “I felt that I had nothing to look forward to.” A sample item for anxiety is as follows: “I felt scared without any good reason.” A sample item for stress is as follows: “I found myself getting agitated.” Items were rated on a 4-point Likert scale ranging from 0 (did not apply to me at all) to 4 (applied to me very much or most of the time). Details of interpreting the scores and the use in this study have been described previously [38].

#### Psychosocial Adaptability

Psychosocial adaptability was an 8-item composite measure (eg, “I was able to adjust my regular social activities to my satisfaction,” “I was able to adjust the way I interact with those I lived with to my satisfaction,” and “I was able to adjust to how I spend my free time [eg, hobbies and entertainment] to my satisfaction”). Participants responded on a 5-point Likert scale ranging from 1 (strongly disagree) to 5 (strongly agree). The internal consistency of the scale was good (α=.82).

#### Social Support

Social support was measured using the Resilience Scale for Adults subscale [39]. Sample items in this 3-item measure included “I have some close friends/family members who really care about me” and “I always have someone who can help me when needed.” Participants responded on a 5-point Likert scale ranging from 1 (strongly disagree) to 5 (strongly agree). The scale has good internal consistency (α=.86).

#### Health Status

Health status was a single-item measure from the 36-Item Short Form Survey (SF-36) [40]. Participants responded to the question “In general, would you say your health is” using the following options: 1 (poor), 2 (fair), 3 (good), 4 (very good), and 5 (excellent).

#### Other Measures

Data on the background characteristics of the participants were collected, including (1) age, (2) gender, (3) marital status, (4) nationality, (5) ethnicity, (6) religion, (7) education level, and (8) occupation.

### Power Analysis

Based on an a priori power analysis (G*Power 3.1.9.7) using a power of 0.80 and an error probability of 0.05, a sample size of 300 participants was required for each group to detect a between-group difference of a small to moderate effect size.

## Results

### Participant Characteristics

A total of 602 participants (Table 1) were recruited for the study (mean age 53.30 years, SD 16.26 years). Of these 602 participants, 302 were categorized as younger (21-59 years old; mean age 39.87 years, SD 11.46 years) and the other 300 were categorized as older (60 years or above; mean age 66.82 years, SD 5.84 years). The majority of the younger adults completed tertiary education (213/302, 70.5%) and were employed (234/302, 77.5%), while the majority of the older adults completed secondary education (145/300, 48.3%) and were largely retired (145/300, 48.3%) or were no longer in employment (44/300, 14.7%).

**Table 1:** Descriptive characteristics of the study sample stratified by age group (younger vs older).

Variable			All adults (N=602)		Younger adults^a^ (n=302)		Older adults^b^ (n=300)
Age (years), mean (SD)			53.30 (16.26)		39.87 (11.46)		66.82 (5.84)
**Sex, n (%)**							
	Male	302 (50.2)		133 (44.0)		169 (56.3)	
	Female	300 (49.8)		169 (56.0)		131 (43.7)	
**Marital status, n (%)**							
	Married	431 (71.6)		188 (62.3)		243 (81.0)	
	Single	124 (20.6)		103 (34.0)		21 (7.0)	
	Divorced	22 (3.7)		11 (3.6)		11 (3.7)	
	Widowed	25 (4.2)		0 (0.0)		25 (8.3)	
**Nationality, n (%)**							
	Singaporean	565 (93.9)		274 (90.7)		291 (97.0)	
	Permanent resident	37 (6.2)		28 (9.3)		9 (3.0)	
**Ethnicity, n (%)**							
	Chinese	404 (67.1)		198 (65.6)		206 (68.7)	
	Malay	91 (15.1)		49 (16.2)		42 (14.0)	
	Indian	82 (13.6)		43 (14.2)		39 (13.0)	
	Other	25 (4.2)		12 (4.0)		13 (4.3)	
**Religion, n (%)**							
	Buddhism	181 (30.1)		95 (31.5)		86 (28.7)	
	Islam	106 (17.6)		58 (19.2)		48 (16.0)	
	Christianity (non-Roman Catholic)	81 (13.5)		34 (11.3)		47 (15.7)	
	Hinduism	60 (10.0)		28 (9.3)		32 (10.7)	
	Roman Catholic	48 (8.0)		15 (5.0)		33 (11.0)	
	Taoism	15 (2.5)		7 (2.3)		8 (2.7)	
	Sikhism	3 (0.5)		2 (0.7)		1 (0.3)	
	No religion	108 (17.9)		63 (20.9)		45 (15.0)	
**Education, n (%)**							
	Primary or below	56 (9.3)		6 (2.0)		50 (16.7)	
	Secondary	200 (33.2)		55 (18.2)		145 (48.3)	
	Postsecondary (nontertiary)	54 (9.0)		28 (9.3)		26 (8.7)	
	Tertiary or above	292 (48.5)		213 (70.5)		79 (26.3)	
**Occupation, n (%)**							
	Employed	345 (57.3)		234 (77.5)		111 (37.0)	
	Not in employment	109 (18.1)		65 (21.5)		44 (14.7)	
	Retired	148 (24.6)		3 (1.0)		145 (48.3)	

^a^Younger adults refer to participants aged 21-59 years.

^b^Older adults refer to participants aged 60 years or above.

### Internet Use Patterns

Up to a third of older adults (102/300, 34.0%) in the study sample reported not using digital media to communicate or run errands during the CB measurement period compared with only 2.7% (8/302) of younger adults who did not do so. Among older adults who did not use the internet (102/300, 34.0%), an overwhelming majority did not have tertiary education (94/102, 92.2%). For older adults, the top 3 reasons for using the internet (Table 2) were social media (154/300, 51.3%), telecommunication (120/300, 40.0%), and online banking (111/300, 37.0%), whereas for younger adults, the top reasons were social media (253/302, 83.8%), online banking (234/302, 77.5%), food delivery (211/302, 69.9%), and telecommunication (210/302, 69.5%).

**Table 2:** Comparison of the frequency of internet use patterns between younger and older adults in the study sample during the COVID-19 pandemic.

Internet use	All adults (N=602), n (%)	Younger adults^a^ (n=302), n (%)	Older adults^b^ (n=300), n (%)
Social media	407 (67.6)	253 (83.8)	154 (51.3)
Online banking	345 (57.3)	234 (77.5)	111 (37.0)
Online telecommunication	330 (54.8)	210 (69.5)	120 (40.0)
Online entertainment	288 (47.8)	189 (62.6)	99 (33.0)
Online food delivery	288 (47.8)	211 (69.9)	77 (25.7)
Online shopping	254 (42.2)	195 (64.6)	59 (19.7)
Online grocery	203 (33.7)	149 (49.3)	54 (18.0)
Did not use	110 (18.3)	8 (2.7)	102 (34.0)

^a^Younger adults refer to participants aged 21-59 years.

^b^Older adults refer to participants aged 60 years or above.

### Use of Resources to Meet Needs

Overall, agreement scores on the ability to use digital platforms to meet needs were lower for older adults (mean 3.37, SD 1.14) than for younger adults (mean 4.17, SD 0.78) (*t*_600_=10.04; *P*<.001; *d*=0.82), and the difference was large in magnitude. The ability to use digital resources for information updates related to the pandemic was lower for older adults (mean 3.28, SD 1.20) than for younger adults (mean 4.22, SD 0.75) (*t*_600_=10.50; *P*<.001; *d*=0.86), and the difference was large in effect size.

More specifically, for older adults who were able to use the internet (Table 3), the agreement scores in their ability to do so were lower compared with the scores for younger adults in the areas of using telecommunication (*t*_427_=7.55; *P*<.001; *d*=0.76), obtaining supplies (*t*_477_=8.97; *P*<.001; *d*=0.82), using online services (*t*_503_=8.17; *P*<.001; *d*=0.74), and changing appointments (*t*_498_=3.53; *P*<.001; *d*=0.31). The effect sizes of the differences were large on the whole, except for the last variable.

**Table 3:** Differences in study sample mean scores between younger and older adults for various essential activities that were conducted over the internet during the COVID-19 pandemic.

Variable	All adults (N=602)			Younger adults^a^ (n=302)			Older adults^b^ (n=300)	
	Observations, n	Score, mean (SD)	Observations, n		Score, mean (SD)	Observations, n		Score, mean (SD)
Buy takeaway	587	4.16 (0.58)	296		4.23 (0.57)	291		4.09 (0.59)
Run errands	590	4.07 (0.69)	298		4.07 (0.75)	292		4.07 (0.62)
Use telecommunication	429	3.98 (1.01)	272		4.25 (0.77)	157		3.53 (1.20)
Change appointments	500	3.86 (0.78)	250		3.98 (0.73)	250		3.74 (0.81)
Use online services	505	3.84 (1.05)	287		4.16 (0.81)	218		3.43 (1.18)
Obtain supplies	479	3.72 (1.09)	272		4.08 (0.83)	207		3.25 (1.20)

^a^Younger adults refer to participants aged 21-59 years.

^b^Older adults refer to participants aged 60 years or above.

### Use of the Internet Before and After the Pandemic

Among older adults, there were minimal changes in the nonuse of the internet for online shopping for essential items (Table 4) before and during or after the pandemic (186/300, 62.0% and 183/300, 61.0%, respectively). This relationship was similar among younger adults, although a much smaller proportion of younger adults did not use the internet for online shopping for essential items before and during or after the pandemic (45/302, 14.9% and 39/302, 12.9%, respectively).

**Table 4:** Differences in the frequency of the use of the internet before and after the COVID-19 pandemic for obtaining essential items as self-reported by younger and older adults in the study sample.

Internet use pattern	All adults (N=602), n (%)	Younger adults^a^ (n=302), n (%)	Older adults^b^ (n=300), n (%)
Use of the internet before the pandemic	371 (61.6)	257 (85.1)	114 (38.0)
Use of the internet after the pandemic	380 (63.1)	263 (87.1)	117 (39.0)

^a^Younger adults refer to participants aged 21-59 years.

^b^Older adults refer to participants aged 60 years or above.

### Time Spent on the Internet

There was a significant negative relationship between time spent on the internet and age (*r*=−0.63; *P*<.001), indicating that increasing age is associated with decreasing time spent on the internet. On average, older adults indeed spent significantly less time on the internet for both work and personal uses per day (mean 146.00 min, SD 9.18 min) compared with younger adults (mean 433.27 min, SD 14.32 min) (*t*_600_=16.84; *P*<.001; *d*=1.38).

### Age, Time Spent on the Internet, and Distress

Zero-ordered bivariate correlations revealed that there was a negative relationship between age and the well-being indicators of depression scores (*r*=−0.36; *P*<.001), anxiety scores (*r*=−0.22; *P*<.001), and stress scores (*r*=−0.19; *P*<.001).

There was also a significant positive association between time spent on the internet and depression scores (*r*=0.31; *P*<.001), anxiety scores (*r*=0.23; *P*<.001), and stress scores (*r*=0.28; *P*<.001). Time spent on the internet was also positively associated with stress concerns (*r*=0.08; *P*=.04).

On interpreting the findings, depression scores suggested that those who spent more time on the internet tended to experience less positive feelings, feeling nothing to look forward to, difficulty in working up initiative, etc. For anxiety scores, those who spent more time on the internet tended to feel worried and scared. Likewise, for stress scores, those who spent more time on the internet tended to experience difficulty in winding down, overreaction to situations, and difficulty in relaxing. The effect sizes for these indicators ranged from low to medium.

### Use of the Internet and Mental Health

Links between internet use patterns in this sample and mental health were explored. The findings showed that mental health was significantly better among those who did not use the internet for social media compared with those who did (Table 5). The effect sizes of the differences however were small (*d*_depression_=−0.27; *d*_anxiety_=−0.17; *d*_stress_=−0.31). This was similarly the case among those who did not use the internet for online shopping compared with those who did (Table 6), with the effect sizes being small (*d*_depression_=−0.44; *d*_anxiety_=−0.17; *d*_stress_=−0.31).

**Table 5:** Differences in depression, anxiety, and stress scores by social media use (did not use vs used) in the study sample using the t-test for equality of means.

Variable	Did not use social media, mean (SD)	Used social media, mean (SD)	*t* (*df*)	*P* value	Cohen *d*
Depression score	8.09 (2.25)	8.82 (2.86)	−3.12 (600)	<.001	−0.27
Anxiety score	7.69 (1.54)	7.99 (1.90)	−1.94 (600)	.03	−0.17
Stress score	8.28 (2.53)	9.18 (2.98)	−3.61 (600)	<.001	−0.31

**Table 6:** Differences in depression, anxiety, and stress scores by online shopping use (did not use vs used) in the study sample using the t-test for equality of means.

Variable	Did not use online shopping, mean (SD)	Used online shopping, mean (SD)	*t* (*df*)	*P* value	Cohen *d*
Depression score	8.09 (2.21)	9.26 (3.13)	−5.37 (600)	<.001	−0.44
Anxiety score	7.74 (1.73)	8.11 (1.87)	−2.51 (600)	<.001	−0.21
Stress score	8.45 (2.52)	9.49 (3.20)	−4.49 (600)	<.001	−0.37

### Main Effects of Time Spent on the Internet and Age on Distress

The main effects of time spent on the internet and age on distress were examined using regression modeling. The regression models were statistically significant for depression (*F*_5,501_=39.64; *P*<.001), anxiety (*F*_5,501_=19.84; *P*<.001), and stress (*F*_5,501_=31.40; *P*<.001). The models explained 28%, 17%, and 24% of the variances in depression, anxiety, and stress, respectively (see Table 7 for the adjusted and unadjusted models).

To facilitate comparison of the effect sizes, all variables were standardized. Examining the variables in the models, time spent on the internet and age were significant predictors of depression, anxiety, and stress. This was the case even after controlling for the effects of individual adaptability, social support, and health status. Standardized regression coefficients showed that the magnitude of the effect of time spent on the internet was higher than that of age for anxiety (β_age_=−0.16; β_time_ _spent on the internet_=0.18) and stress (β_age_=−0.17; β_time_ _spent on the internet_=0.21), but this effect was lower for depression (β_age_=−0.24; β_time_ _spent on the internet_=0.20). Overall, the findings suggested that distress was associated with decreasing age, and likewise, this was the case for those who spent more time on the internet.

To examine if the effect of age on mental health was influenced by time spent on the internet, mediation analysis was conducted using the approach advocated by Baron and Kenny [41]. The bootstrapping method with 1000 resamples to estimate the 95% CI was additionally conducted to investigate the significance of indirect effects [42]. A significance level of *P*<.05 was used for all analyses.

**Figure 1:**
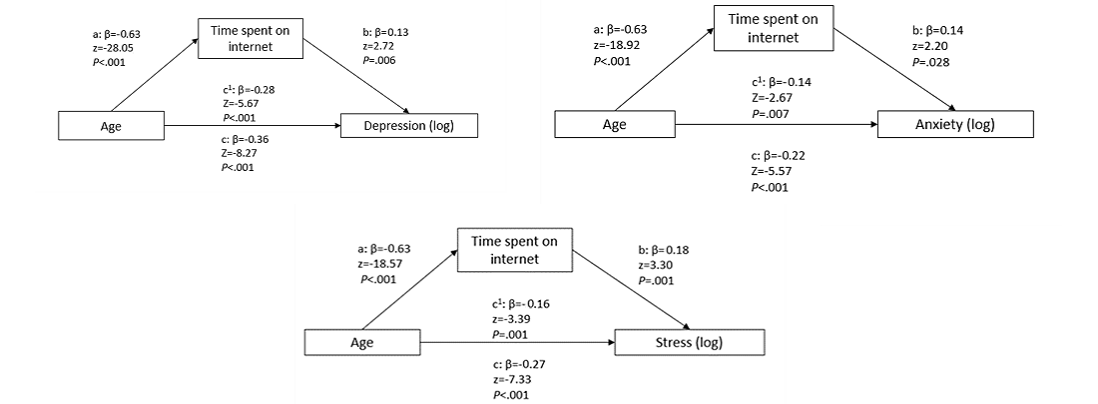
Mediation models illustrating the partial mediating role of time spent on the internet for the effect of age on mental health (mental health was indexed by depression, anxiety, and stress individually). All presented effects are standardized. The a-path is the direct effect, b-path is the direct effect, c1-path is the direct effect, and c-path is the total effect.

To facilitate interpretation of the effect sizes, all variables were standardized to reflect the strength of correlations. Results on the partial mediating role of time spent on the internet for the effect of age on mental health are shown in Figure 1.

In all models, mediation analyses showed that there was a significant indirect effect of age on mental health through time spent on the internet. For depression, the indirect effect of time spent on the internet was as follows: *ab*=−0.08; *z*=−2.66; *P*=.008. The mediation effect accounted for 23% of the total effect (β=−0.36). For anxiety, the indirect effect of time spent on the internet was as follows: *ab*=−0.09; *z*=−2.15; *P*=.03. The mediation effect was moderate and accounted for 39% of the total effect (β=−0.24). For stress, the indirect effect of time spent on the internet was as follows: *ab*=−0.11; *z*=−3.20; *P*=.001. The mediation effect was moderate and accounted for 42% of the total effect (β=−0.22). Examining the *r*^2^ of the models, all variables together explained 43% of the variance in depression, 40% of the variance in anxiety, and 40% of the variance in stress.

Overall, the effect of age on mental health was partially explained by time spent on the internet. On interpreting the *r*^2^ of the models and effect sizes, a conclusion could be drawn that the mediating role of time spent on the internet for the effect of age on mental health was relatively substantial, especially for anxiety and stress. However, as full mediation did not occur, for time spent on the internet, which played a substantial role, the effect through age was not a major causal pathway.

**Table 7:** Main effects of time spent on the internet and age on distress using multivariate regression analysis.

Variable	Depression scores									Anxiety scores									Stress scores						
	Unadjusted Model 1				Adjusted Model 1^a^				Unadjusted Model 1					Adjusted Model 1^a^				Unadjusted Model 1					Adjusted Model 1^a^		
	Β	*t* (*df*)	*P* value	β		*t* (*df*)	*P* value	β			*t* (*df*)	*P* value	β		*t* (*df*)	*P* value	β			*t* (*df*)	*P* value	β		*t* (*df*)	*P* value
Age	−0.28	−5.68 (602)	<.001	−0.24		−4.97 (507)	<.001	−0.14			−2.71 (602)	.007	−0.16		−3.03 (507)	.003	−0.16			−3.21 (602)	.001	−0.17		−3.39 (507)	.001
Time spent on the internet	0.13	2.76 (602)	.006	0.20		4.03 (507)	<.001	0.007			2.72 (602)	<.001	0.18		3.39 (507)	.001	0.18			3.63 (602)	<.001	0.21		4.31 (507)	<.001

^a^Adjusted for adaptability, social support, and health status.

## Discussion

### Principal Findings

This study examined the relationship among age, internet use, and mental health during the lockdown phase of the COVID-19 pandemic. Specifically, we compared internet use between younger and older adults and examined the mediating role of internet use in the relationship between age and mental health. In support of our hypothesis, older adults were reported to spend less time on the internet as compared with their younger counterparts. In addition, internet use was found to partially mediate the effects of age on depression, anxiety, and stress.

Given that ICT is ubiquitous in today’s world, digital exclusion of older adults is emerging as an imperative concern amid rapidly aging populations. Previous studies have reported poor adoption of ICT among older adults [43-46], but findings specific to older adults during the early phase of the COVID-19 pandemic were sparse. The findings of this study extend this empirical evidence by demonstrating that such an age-related digital divide was pervasive during the COVID-19 pandemic. In support of our hypothesis, internet use among older adults during the lockdown period was found to be less as compared with that among their younger counterparts. Specifically, the use of digital platforms for essential services and entertainment purposes was less prevalent among older adults than among younger adults. While age has been known to be a predictor of internet use [47,48], the large effect size (*d*=1.38) found in this study suggests that the magnitude of such a grey digital divide is substantial and warrants more attention. Given that higher education is also associated with greater internet use [47], our results may be confounded by lower education levels among older adults in the study sample. Such a difference may also be plausibly attributed to the negative relationship between age and technology acceptance [49] or fear of stereotype threats regarding technology use in older adults [50].

Imposed spatial distancing measures during the COVID-19 pandemic have proliferated the adoption of technology to help individuals meet essential needs and stay connected with one another. This has been claimed to amplify the digital divide between younger and older adults [15,51,52]. Indeed, our findings showed that older adults not only used less internet than their younger peers, but also reported to be less able to use online services to run essential activities during the lockdown phase of the pandemic. This suggests that older adults were less capable of adapting to digital means to meet their basic needs. Our findings corroborate the results of another study reporting that the inability of older adults to use digital devices limited their access to transportation, medical care, and food supplies during the pandemic [52]. Consequently, this has raised concerns that the negative impacts of the pandemic on mental health may be disproportionate in certain groups, such as older adults, who may risk being excluded from the society due to such a digital divide [53].

While lockdown measures were well intended to mitigate infections during the COVID-19 pandemic, they had detrimental effects on the psychological well-being of individuals [5]. The results of this study suggest that both age and internet use are significant predictors of mental health, and importantly, such associations persisted even after accounting for adaptability, social support, and health status. As purported by the socioemotional selectivity theory [54], we found that older age was associated with better mental health. Specifically, we previously reported that older adults had lower depression, anxiety, and stress levels as compared with younger adults [38]. Our results are consistent with those of other studies that reported better mental health in older adults during the pandemic as compared with younger adults [54-57]. In contrast, higher internet use during the lockdown period was found to be associated with poorer mental health. While digital technologies certainly could aid individuals in the continuation of their work and daily essential activities during the lockdown, they could also be used in a disordered manner [17,18]. Our results suggest that it could be the case of the latter since mental distress has been observed to be higher in those with higher internet use (ie, spent more time on the internet and used more social media and online shopping). Indeed, the risk of problematic internet use was reported to have increased during the pandemic [2].

Many studies conducted during the earlier phase of the COVID-19 pandemic focused on the effects of internet use on younger adults and adolescents who were often conveniently sampled [25,28,30-32]. In this study, a door-to-door survey using a stratified random sampling method ensured that potential sampling bias issues were circumvented. This study ensured that there were adequate responses sampled from participants in the older age group (ie, over 60 years of age) and therefore contributed to the literature by showing the effects of internet use in not only younger adults but also older adults. This study was therefore able to address calls [34] for research comparing the linkages among age, internet use, and mental well-being during the COVID-19 pandemic. Importantly, our statistical modeling suggests that internet use acts as a partial mediator for the effects of age on depression, anxiety, and stress levels. This suggests that the inverse relationship between age and mental distress could be partially explained by the amount of time spent on the internet by younger adults. As mentioned earlier, younger adults were found to spend more time on the internet as compared with older adults. Such increased use may be problematic and consequently have negative effects on mental health. Indeed, problematic internet use during the COVID-19 pandemic has been reported to be associated with poorer mental well-being [58,59]. Studies have reported that pandemic-related stress is associated with tendencies toward problematic digital use [59-61]. Given that younger adults were found to have greater stress concerns during the pandemic [38], it may be the case that younger adults experienced greater stress and thus engaged in greater problematic internet use, which resulted in poorer mental health. More research is recommended to investigate this. Our findings support the recommendations to mitigate the risk of problematic internet use during the pandemic [2].

Despite the negative relationship between internet use and mental health, it is important to note that not all types of digital use had detrimental consequences on mental health, and ICT use for the right purposes could be potentially beneficial. For example, a recent scoping review revealed that internet use for communication purposes was associated with better mental health for older adults during the COVID-19 pandemic [62]. Indeed, older adults have reported the importance of ICT to help them maintain their social connections during the lockdown period [63-65]. This study showed that social media use was the highest among the various uses of the internet even for older adults, and a future study can consider investigating how older adults use social media and assessing the benefits they derive from it during the pandemic. Given that some older adults may experience loneliness due to lack of physical contact, especially under the exceptional circumstances imposed by the pandemic, the use of ICT can help to allay the negative effects of social isolation. Thus, in view of the large digital divide between younger and older adults found in this study, greater efforts are necessary to close such a divide, including advocating for age-sensitive design of technologies [49,52] and deterring stereotype threats associated with technology use [50]. Considering the potential negative relationship between internet use and mental health, it is also imperative to educate older adults in using ICT appropriately.

### Limitations

Notwithstanding the contributions of this study to existing literature, it is important to acknowledge some limitations. First, the study sample consisted of community-dwelling adults, and thus, the findings may not be generalizable to specific subgroup populations who may be more vulnerable. The relationship between age and mental distress observed in this study may differ if other groups of older adults are included, such as those residing in nursing homes or experiencing diminished mental capacity.

Second, the interpretation of this study’s results is limited by its outcome measures. Although this study established the partial mediating role of internet use for the effects of age on depression, anxiety, and stress, the study did not collect measures that would be important indicators of disordered use of the internet in younger adults, including online gambling, viewing pornography, and playing video games. Nonetheless, there is evidence from various studies indicating that increased time spent on the internet during the earlier phase of the COVID-19 pandemic was the main contributory factor for a number of mental health problems, such as depression, anxiety, and stress [66-68]. The findings from this study are therefore aligned with the existing research and add to this field by presenting data from a developed country with high digital adoption rates. However, a future study can consider examining how the pandemic could have possibly exacerbated problematic internet use and whether time spent on the internet is indeed an adequate proxy. The digital divide and its relationship with mental health can be influenced by a myriad of factors going beyond problematic internet use. There is a possibility that increased internet use in younger adults during the lockdown period was attributed to having to work remotely from office, and the distress experienced could therefore be attributed to this new form of work arrangement rather than problematic internet use per se. Given that the study was making reference to the first lockdown period, younger adults may not have adapted to this form of working arrangement despite the known benefits of ICT, including continuation of their work and running of other essential activities.

Finally, all measures of this study were collected from participants’ retrospective recollection of their experiences during the lockdown period. Since time spent on the internet was not based on any objective indicator, we cannot rule out the possibility that this or other attitudinal responses are subject to recall bias.

### Conclusion

This study showed that older adults lagged behind younger adults in the use of digital resources during the pandemic, which could have helped them in communication and socialization, and the findings support existing literature on the poor adoption of ICT among older adults. This study further contributes to the literature by showing how through mediation modeling, the negative relationship between increasing age and mental distress appears to be partially explained by the amount of time spent on the internet by younger adults. Without moderate use, the benefits brought by digital technologies could have been attenuated during the lockdown phase of the pandemic. It is imperative to educate both young and old adults in the appropriate use of ICT.

## Abbreviations

AMT: Abbreviated Mental Test
CB: circuit breaker
DASS-21: Depression, Anxiety, and Stress Scale
ICT: information and communication technology

## Data Availability

The data sets used or analyzed during this study are available from the corresponding author on reasonable request. The data sets used cannot be shared openly as the participants have not consented to share their data openly, and this is therefore not allowed under the approved study protocol.
